# Interrater reliability in the assessment of physiotherapy students

**DOI:** 10.1186/s12909-022-03231-y

**Published:** 2022-03-16

**Authors:** Flora P. Gittinger, Martin Lemos, Jan L. Neumann, Jürgen Förster, Daniel Dohmen, Birgit Berke, Anke Olmeo, Gisela Lucas, Stephan M. Jonas

**Affiliations:** 1 Department of Medical Informatics, Faculty of Medicine, RWTH Aachen University, Pauwelsstraße 30, 52074 Aachen, Germany; 2 Audiovisual Media Center, Faculty of Medicine, RWTH Aachen University, Aachen, Germany; 3 Schule für Physiotherapie, Uniklinik RWTH Aachen, Aachen, Germany; 4 Berufsfachschule für Physiotherapie, Grone-Bildungszentrum für Gesundheits- und Sozialberufe GmbH, Hamburg, Germany; 5 Department of Informatics, Technical University of Munich, Munich, Germany; 6 Department of Digital Health, University Hospital Bonn, Bonn, Germany

**Keywords:** Interrater reliability, Physiotherapy education, Assessment of clinical skills

## Abstract

**Background:**

Reliable and objective assessment of psychomotor skills in physiotherapy students’ education is essential for direct feedback and skill improvement. The aim of this study is to determine the interrater reliability in the assessment process of physiotherapy students and to analyse the assessment behaviour of the examiners.

**Methods:**

Physiotherapy teachers from two different schools assessed students from two different schools performing proprioceptive neuromuscular facilitation (PNF) patterns. An evaluation sheet with a 6-point rating scale and 20 evaluation criteria including an overall rating was used for assessment. The interrater reliability was determined calculating an intraclass-correlation coefficient (ICC) and Krippendorff’s alpha. The assessment behaviour of the examiners was further analysed calculating the location parameters and showing the item response distribution over item in form of a Likert plot.

**Results:**

The ICC estimates were mostly below 0.4, indicating poor interrater reliability. This was confirmed by Krippendorff’s alpha. The examiners showed a certain central tendency and intergroup bias.

**Discussion and conclusion:**

The interrater reliability in this assessment format was rather low. No difference between the two physiotherapy schools concerning the interrater reliability could be identified. Despite certain limitations of this study, there is a definite need for improvement of the assessment process in physiotherapy education to provide the students with reliable and objective feedback and ensure a certain level of professional competence in the students.

**Trial registration:**

The study was approved by the ethics committee of the Medical Faculty RWTH Aachen University (EK 340/16).

**Supplementary Information:**

The online version contains supplementary material available at 10.1186/s12909-022-03231-y.

## Background

Reliability and validity in assessing clinical or practical skills is a continuous challenge in the education of health care professionals such as physiotherapists [1].

The term “clinical skills” is not unanimously defined. The definitions include different aspects such as practical procedures and communication skills among others [2]. They are also referred to as psychomotor skills or procedural skills [3]. In the following, the term psychomotor skills will be used, as the focus of this study lies on manual tasks performed by physiotherapists within therapeutical interventions.

Almost every discipline in the health care sector has to face the challenge of teaching psychomotor skills [3]. Health professions strongly identify themselves [2] with their psychomotor skills and each profession has their own unique set of skills [4]. In medical education, residents must learn psychomotor skills from comparably small procedures, such as venepuncture, to complex procedures, such as cardiac catheterisation in cardiology. The practice of these psychomotor skills has a relevant influence on the job satisfaction [5] .

The same importance of psychomotor skills applies for the physiotherapy profession, where the taught skills are complex. One skill consists of a variety of single components, which must be executed simultaneously to achieve the required effect in the patient’s treatment [6]. To impart and assess these skills is a substantial part in the education of physiotherapy students.

In the last decades, there have been ambitions in the community of physiotherapists in Germany to standardize the assessment process of these skills, for example, by using the mini-Clinical Evaluation Exercise (mini-CEX) assessment format. The mini-CEX has already proven to be a valid and reliable tool for assessing medical students [7].

A key issue in the assessment process is the objectivity of examiners and the risk of examiner bias [8]. The observation-based assessment is usually of a subjective nature [9]. It is crucial to have reliable measurements of students’ performance to be able to make decisions about their competence and fitness to practice and to give them reliable feedback on how to improve their skills [1].

The training as a physiotherapist in Germany lasts 3 years and is completed with a state examination. Psychomotor skills are essential to the education and an integral part of the mandatory coursework [10]. Learning a psychomotor skill--the encryption in the motor cortex--is reliant upon both skill practice opportunities and terminal feedback [4]. Thus, it is necessary to provide this feedback [11] in the most objective way possible.

To give insights into the current status of the quality of this feedback, the aim of this study is to assess the interrater reliability of the evaluation of psychomotor skills in the education of physiotherapy students based on an assessment tool originally created for peer evaluation (mini-PEX) in lieu of a mini-CEX evaluation. In addition, we took a brief look at the examiners’ assessment behaviour.

This study is part of the “Media Didactics Meets Wearable Computing (MediWeCo)” project (project number FKZ 01PD15013) founded by the German Federal Ministry of Education and Research (BMBF) through the program “Digital media in vocational education” supported by the European Union Social Fund (ESF). MediWeCo aims to develop digital learning alternatives to support and improve the process of teaching, learning, and assessing psychomotor skills in physiotherapy.

## Methods

We conducted a study to determine the interrater reliability of the evaluation process in physiotherapy education and to analyse the examiners’ assessment behaviour. Specifically, physiotherapy teachers evaluated physiotherapy students performing proprioceptive neuromuscular facilitation (PNF) patterns. PNF is a widely used treatment by physiotherapists and forms part of the curriculum in many countries [12]. After informed consent, physiotherapy students were video recorded during their performance and evaluated by the physiotherapy teachers based on the video recording. The evaluation process was standardized with an evaluation sheet (see section “Evaluation” for details) that was introduced to the teachers by means of a short briefing video prior to the evaluation.

The study was approved by the ethics committee of the Medical Faculty RWTH Aachen University (EK 340/16). A total of three examination dates was scheduled, two at the “Schule für Physiotherapie Uniklinik RWTH Aachen” (AC) and one at the “Grone Berufsfachschule für Physiotherapie Hamburg” (HH).

### Participants and data

A total of 47 students participated in the trial, 21 and 26 from HH and AC respectively. The physiotherapy students were in their first year of training and were instructed in PNF. All students gave their written consent to participate in this study. The AC students had two examination dates with a total of 41 exams as AC students were absent on the day of the examination in 11 cases. The HH students had one examination date with 21 examinations. Each examination was video recorded with a total of 62 videos.

### Examiners

Six physiotherapy teachers participated in this study, three from HH and three from AC. The six physiotherapy teachers were anonymised and numbered consecutively from 1 to 6, with AC teachers having numbers 1 to 3 and HH teachers having numbers 4 to 6.

### PNF patterns

Three PNF leg patterns were selected: 1) extension-abduction-internal rotation with knee extension, 2) extension-adduction-external rotation with knee extension, 3) flexion-adduction-external rotation with knee flexion.

The first two patterns were performed by the AC students, the third by the HH students. We chose different patterns for the two schools to avoid competition and direct comparison between the two schools in hopes of minimizing intergroup bias. All three patterns were executed using the technique Rhythmic Initiation consisting of four phases: Passive, Passive-Active, Resistive, and Active phase. Each phase of this technique was repeated 10 times.

### Evaluation

The video recordings of the students’ performance were evaluated afterwards by four examiners out of the group of six examiners. The four examiners were chosen randomly for every single performance, two out of the three from HH and two out of the three from AC. Because of the workload it was not possible for every examiner to evaluate every performance. The order in which the video recordings were evaluated was not defined. For the evaluation, the examiners used an evaluation sheet developed within the MediWeCo project, consisting of 20 evaluation criteria. The 20 criteria consist of 19 single criteria and one overall rating criterion. The evaluation sheet was originally designed as a peer evaluation tool, a so called mini-Peer Evaluation Exercise (mini-PEX) and used at the “Schule für Physiotherapie Uniklinik RWTH Aachen” (AC). We used it as a mini-CEX evaluation tool. A publication concerning the validity and reliability of this tool is pending. It was not the aim of this study to assess the validity of the evaluation tool. Each evaluation criterion was rated on a six-point Likert scale from 1 to 6, with 6 corresponding to a completely fulfilled criterion. The 20 evaluation criteria are: Bench Height, Treatment Area, Patient Position, Verbal Communication, Explanation, Rhythm, Passive, Active-Assistive, Resistive, Active, End Position, Diagonal, Movement Components, Timing, Body Position, Body Mechanics, Lumbrical Grip, Stimulus, Resistance, Overall Rating.

### Data analysis

To determine the interrater reliability between the examiners, the intraclass correlation coefficient (ICC) was calculated. The ICC is a standard tool to determine the interrater reliability of more than two raters on the basis of interval scaled data. The six-point rating scale used in this study is interval-scaled.

To compare the single ratings from each examiner, ICC estimates and their 95% confidence intervals were calculated using IBM SPSS Statistics for Macintosh, Version 25.0. (Armonk, NY: IBM Corp), based on a single-rating, absolute agreement, one-way random effects model (ICC 1,1) [13–15]. To compare AC and HH examiners, ICC estimates and their 95% confidence intervals were calculated on the mean rating of the AC examiners and the mean rating of the HH examiners, based on a mean-rating, absolute agreement, one-way random effects model (ICC 1,k).

For the single ratings, 15 ICC estimates were calculated, one between examiner 1 and 2, 1 and 3, and so on until examiner 5 and 6. Each ICC was calculated separately for every evaluation criterion. Table 1 shows an index of the abbreviations used for the different ICC estimates. The ICC estimates were interpreted using the guidelines suggested by Cicchetti [16]. The ICC estimates are categorized in poor, fair, good, and excellent interrater agreement (Table 2).

**Table 1:** Index of abbreviations for ICC estimates

Examiners	2	3	4	5	6	Mean Ratings AC
1	ICC 1,2 _criterion_	ICC 1,3 _criterion_	ICC 1,4 _criterion_	ICC 1,5 _criterion_	ICC 1,6 _criterion_	
2		ICC 2,3 _criterion_	ICC 2,4 _criterion_	ICC 2,5 _criterion_	ICC 2,6 _criterion_	
3			ICC 3,4 _criterion_	ICC 3,5 _criterion_	ICC 3,6 _criterion_	
4				ICC 4,5 _criterion_	ICC 4,6 _criterion_	
5					ICC 5,6 _criterion_	
Mean Ratings HH						ICC AC vs HH _criterion_

**Table 2:** Guidelines interpreting ICC estimates by Cicchetti

ICC Estimates	Meaning
*< 0.40*	poor interrater agreement
*0.40–0.59*	fair interrater agreement
*0.60–0.74*	good interrater agreement
*0.75–1.00*	excellent interrater agreement

To verify the ICC estimates, we additionally calculated Krippendorff’s alpha. Krippendorff’s alpha was interpreted by the guidelines suggested by Krippendorff, with α ≥ 0.800 indicating good interrater reliability, 0.800 > α ≥ 0.667 only allowing for tentative conclusions, and α <  0.667 suggesting poor interrater reliability [17].

To analyse the examiners’ assessment behaviour and to illustrate the distribution of ratings from AC and HH, we calculated the location parameters for the Overall Rating for the two groups (AC and HH) of examiners and students and visualized the data in form of box plots (Fig. 4). Additionally, the score distribution is shown in the form of a Likert plot.

## Results

### Intraclass correlation coefficient and Krippendorff’s alpha

**Fig. 1:**
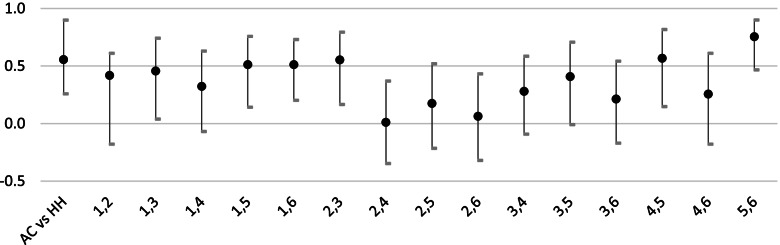
ICC Estimates with 95% confidence interval for the criterion Overall Rating

The ICC estimate comparing the mean rating of the AC examiners and the mean rating of the HH examiners for the criterion Overall Rating (ICC AC vs HH _Overall Rating_) is 0.559 (95% confidence interval, CI 0.258–0.899) (Fig. 1). This signifies a fair interrater agreement between the two groups of examiners. The ICC 5,6 _Overall Rating_ estimate has the highest value of 0.755 (CI 0.466–0.9) in the category Overall Rating signifying an excellent interrater agreement between examiner 5 and 6. The ICC 2,4 _Overall Rating_ estimate has the lowest value of 0.011 in the category Overall rating (CI -0.347 – 0.369) signifying a poor interrater agreement.

**Fig. 2:**
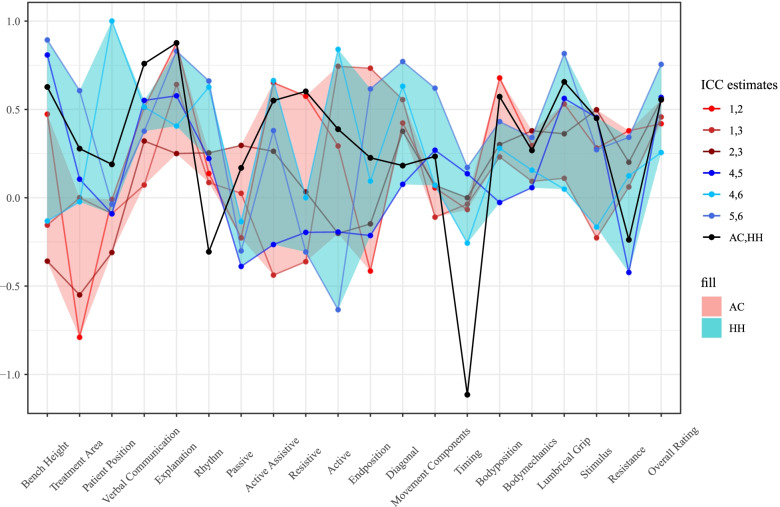
ICC estimates AC vs HH examiners, ICC estimates AC examiners, ICC estimates HH examiners. Note: red = AC examiners, blue = HH examiners, black = AC vs HH examiners

Comparing the ratings of the HH examiners and AC examiners, no systematic difference in the ICC estimates was obvious (Fig. 2). The ICC estimates within the group of HH examiners (ICC 4,5; ICC 4,6; ICC 5,6) or within the group of AC examiners (ICC 1,2; ICC 1,3; ICC 2,3) are not higher than the ICC estimates comparing AC and HH examiners (ICC AC vs HH).

In the group of AC examiners (ICC 1,2; ICC 1,3; ICC 2,3) 44 (73.3%) of the altogether 60 ICC estimates fall into the category poor, 11 (18.3%) into the category fair, 4 (6.7%) into the category good, and 1 (1.7%) into the category excellent. In the group of HH examiners (ICC 4,5; ICC 4,6; ICC 5,6) 37 (61.7%) of the altogether 60 ICC estimates fall into the category poor, 8 (13.3%) into the category fair, 7 (11.7%) into the category good, and 8 (13.3%) into the category excellent. Comparing AC and HH examiners (ICC AC vs HH) 11 (55%) of the altogether 20 ICC estimates fall into the category poor, 4 (20%) into the category fair, 3 (15%) into the category good, and 2 (10%) into the category excellent.

Overall, the ICC estimates vary between poor and excellent depending on which criterion is rated and by whom it is rated, but with the estimates indicating poor interrater reliability being predominant. An additional table shows this in more detail (see Additional file 1). In absolute numbers, 229 of the 320 ICC estimates fall into the category poor, which makes a share of 71.6%.

**Fig. 3:**
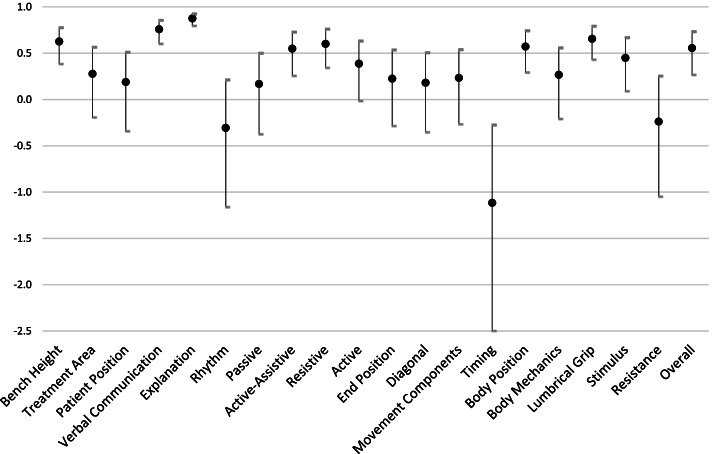
ICC estimates AC vs HH with 95% confidence interval

For example, the ICC estimates comparing the AC and HH examiners (ICC AC vs HH) signify a poor interrater reliability for 11 evaluation criteria (Treatment Area, Patient Position, Rhythm, Passive, Active, End Position, Diagonal, Movement Components, Timing, Body Mechanics, Resistance), a fair interrater reliability for 4 evaluation criteria (Active-Assistive, Body Position, Stimulus, Overall Rating), a good interrater reliability for 3 evaluation criteria (Bench Height, Resistive, Lumbrical Grip), and an excellent interrater reliability for 2 evaluation criteria (Verbal Communication, Explanation) (Fig. 3).

The Krippendorff’s alpha estimates confirm the findings of the ICC estimates. They also indicate a poor interrater reliability for most evaluation criteria and show no systematic difference between the two groups of examiners. The Krippendorff’s alpha estimates are shown in detail in Additional file 2.

### Distribution of ratings

**Fig. 4:**
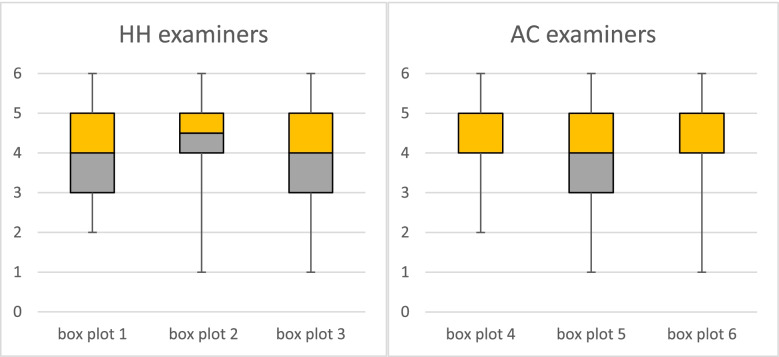
Box plots for ratings by HH and AC examiners. Box plot 1: AC students rated by HH examiners. Box plot 2: HH students rated by HH examiners. Box plot 3: all students rated by HH examiners. Box plot 4: AC students rated by AC examiners. Box plot 5: HH students rated by AC examiners. Box plot 6: all students rated by AC examiners

Looking at the distribution of ratings for the Overall Rating from the two different groups of examiners (Fig. 4), one can see that the students rated by examiners from the same school (HH students rated by HH examiners and AC students rated by AC examiners) tend to get a better rating.

The median for HH students rated by HH examiners is 4,5 (box plot 2), whereas the median for AC students rated by HH examiners is 4 (box plot 1). The lower quartile for HH students rated by HH examiners (box plot 2) is also higher than for AC students rated by HH examiners (box plot 1). However, the minimum for HH students rated by HH examiners (box plot 2) is one point lower than for AC students rated by HH examiners (box plot 1).

The median for AC students rated by AC examiners and HH students rated by AC examiners is the same, but the lower quartile and the minimum for AC students rated by AC examiners is higher than for HH students rated by AC examiners (box plot 4 and 5).

The interquartile range varies between 1 (box plots 2, 4 and 6) and 2 (box plots 1, 3 and 5), meaning that 50% of the ratings lie in the range between 4 and 5, and 3 and 5 respectively. In box plot 4 and 6 the range between lower quartile and median is equal zero, meaning the 25 and 50 percentiles are equal.

Minimum and maximum are equal 1 and 6, respectively, in box plots 2, 3, 5 and 6. Looking at the ratings of AC students given by HH examiners and by AC examiners minimum and maximum are equal 2 and 6, respectively (box plots 1 and 4). The worst rating given to AC students by AC examiners and by HH examiners is one point higher than the worst rating given to HH students by AC examiners and by HH examiners (box plots 1,2,4 and 5). The best rating given is the same no matter which students are rated or by whom they are rated.

The Likert plot (Additional file 3) shows that the good ratings for the single criteria with 3 to 6 points on the rating scale strongly outweigh the bad ratings except when the criteria Verbal Communication and Explanation are considered. Looking at the Overall Rating mostly average to good scores are awarded.

## Discussion

Our results show that the ICC estimates, indicating the interrater reliability, fall predominantly into the category poor interrater agreement (Table 2). Therefore, the ICC estimates suggest that the interrater reliability in this study for the chosen assessment format is rather low.

To the best of our knowledge, no similar studies regarding the interrater reliability in the assessment of physiotherapy students in Germany exist to confirm or contradict our findings.

### Interrater reliability indicated by ICC and Krippendorff’s alpha

This study shows that the interrater reliability is rather low as most ICC estimates (71.6%) lie beneath the mark of 0.4 (see Addendum A for details). There is no strong evidence that the interrater reliability is higher if all examiners are from the same school, or that it is lower if the student is evaluated by examiners from different schools. There seems to be no systematic difference in the way of rating between the two participating schools, but among HH examiners the ICC estimates in the category excellent and good are more frequent than among AC examiners. The HH examiners seem to have a slightly more homogenous way of rating, especially raters 5 and 6 as indicated by the high number of fair to excellent ICC estimates (Additional file 1). One explanation could be a close collaboration between two of the HH examiners, who usually work together for the assessment during the physiotherapy state examination. Due to the anonymisation of the examiners, this could not be verified. Still, also within the HH examiner group, most ICC estimates, namely 61.7%, fall into the category poor.

The Krippendorff’s alpha estimates confirm these findings. They generally show poor interrater reliability, with slightly higher estimates within the group of HH examiners.

This shows that the assessment process does not provide the students with the reliable feedback they need to improve their performance.

Similar results with ICC estimates ranging from 0.14–0.44 were found in a study examining the interrater reliability in the evaluation of clinical skills in neurology using an assessment format comparable to the mini-CEX [18]. Here the interrater reliability between local faculty examiners and examiners of the American Board of Psychiatry was determined by calculating ICC estimates. The ICC estimates in this study also indicated a rather low interrater reliability.

The evaluation criterion with the most acceptable ICC estimates (category fair, good, and excellent) is the criterion Explanation. The examiners’ expectation of the students seems to be very similar concerning this evaluation criterion, whereas the concept of what is a good or bad performance seems to differ strongly for the criteria Passive, Timing, and Resistive. All ICC estimates for these three evaluation criteria fall into the category poor. One could assume, that verbal performance like the criterion Explanation is easier to assess objectively and reliably than motoric performance like the criteria Passive, Timing, and Resistive.

In the end, the rating that a physiotherapy student receives for his/her performance seems to be heavily dependent on which examiner is rating the student’s performance. But a positive aspect is that the interrater reliability seems not to be dependent on which school or location the examiner is from. Yet, our results suggest that the interrater reliability is in high need of improvement.

### Rating distribution

The boxplots comparing the scores for the Overall Rating from HH and AC examiners show a certain intergroup bias or favouritism (Fig. 4). HH examiners give HH students a better rating than they give AC students and AC examiners give AC students a better rating than they give HH students. It is a well-known phenomenon in the field of social psychology that human beings show preferential behaviour towards members of their own group, so called in-groups [19]. Therefore, our results are not surprising.

The better ratings of in-group students may also be caused by the difference in expectations and style of teaching. HH students are taught by HH examiners and know which performance they expect and the same applies for AC students. Students may be able to fulfil the particular expectations of their own examiners more completely than the unknown expectations of external examiners.

Another aspect seen in the box plots (Fig. 4) concerning the Overall Rating is the central tendency bias. The interquartile range varies between 1 and 2 and for two boxplots the range between lower quartile and median is equal 0. The Likert plots (Additional file 3) show that the rating scores on the middle and upper part of the rating scale are predominant for most of the evaluation criteria. The examiners rather give an average or good rating than use the extreme points on the lower part of the rating scale. Besides the examiners tend to give better ratings for the single criteria than for the Overall Rating. For the Overall Rating a central tendency bias can be observed, as mentioned above.

An explanation for this rating behaviour could be the homogeneity of the group of participants. The students are all in their first year of training and on the same skill level, therefore it could be that most of their performances truly lie in the middle of the rating scale. The clustering of ratings in the middle does not necessarily have to be caused by central tendency bias.

### Limitation: interpreting the intraclass correlation coefficient

When interpreting ICC estimates, some pitfalls have to be considered. There are some limitations to the validity of the ICC estimates in this study, one being the relatively small group of participants of 47 physiotherapy students. But as a thumb rule it is suggested that 30 participants are deemed sufficient in reliability studies [15].

In addition, not every physiotherapy student was evaluated by all 6 physiotherapy teachers. One can discuss, that if every teacher would have assessed every student’s performance, this would have led to more data and a potentially more significant ICC estimate, as an ICC estimate comparing ratings of all 6 teachers could have been calculated using a “consistency” [15] or respectively “just” ICC model [20]. This consistency model takes the raters’ bias into account, if the rater is rather strict or lenient. But because of the burden for the examiners, the assessment of every physiotherapy student by every single physiotherapy teacher was not feasible in our case.

The ICC model we used was a one-way random effects, absolute agreement, single rater and multiple rater model, which usually produces lower ICC estimates than the consistency model mentioned above [21]. This has to be taken into consideration when interpreting the ICC estimates, because the actual interrater reliability could be underestimated by using the one-way random affects, absolute agreement model. The one-way random effects, absolute agreement model had to be chosen as not every physiotherapy student was evaluated by the same set of examiners [22].

In addition, decisions about the professional competence, for example, which applicant gets the job, are usually made based on the absolute values of the rating and do not consider differences in the mean values of different raters. This is another reason why the absolute agreement model seems to be more appropriate in this context.

As we used a single and multiple rater model, with the multiple rater model based on mean values from the HH examiners and mean values from the AC examiners, one also has to be aware of the fact that a multiple rater model generates higher ICC estimates than a single rater one [20]. This could have the effect that the difference in the ratings between HH and AC examiners are not shown accurately in our calculations.

Another factor leading to poor interrater reliability could be the lack of variance within the group of physiotherapy students. If the group of participants is too homogeneous in their skills, the ICC estimates underrate the true interrater reliability [20]. This could be a restriction for this study as the physiotherapy students were all in their first year of training.

### Ways to improve reliability in assessment

The existing literature suggests that the training or briefing of the participating raters is one possible way to influence the outcome of interrater reliability. Some sources suggest that rater training improves interrater reliability [23–25], whereas other studies could not find a significant difference if the raters are trained or not [26, 27]. In the presented study, the physiotherapy teachers received a short briefing concerning the evaluation sheet. Further studies are needed to decide whether more extensive rater training will improve the ICC estimates.

Using different assessment formats could be an additional way to improve interrater reliability and make the rating process more objective. The Objective Structured Clinical Examination (OSCE) was introduced in the 1970s by Harden [28] and is widely accepted in medical education nowadays [29–31]. There are some points that must be kept in mind regarding the reliability of OSCEs. The number of stations, which the students rotate round, has to be sufficiently large (in our case the number of different PNF patterns that are examined), the rating strategy and checklists used should be standardized and discussed in advance, and standardized patients should be used preferably [32, 33].

Aside from the OSCE format, standardized assessment tools like the “Assessment of Physiotherapy Practice” tool (APP) could be another possibility to increase reliability, but they are mostly used for an assessment over a longer period of time in clinical settings [34, 35] .

Finally, smart wearables with movement sensors could support the assessment process of psychomotor skills and increase its objectivity not only in the field of physiotherapy. Some sources showed the successful application of smart wearables in the training and evaluation of hand hygiene [36], knee arthroscopy [37], and also physiotherapy education [38]. But this form of assessment is still in its early stages of development and more efforts have to be made to make it useable in real life assessments of psychomotor skills in physiotherapy education and other health care professions.

## Conclusion

Despite all restrictions concerning the ICC estimates, this study still shows a mostly poor interrater reliability in the assessment process in physiotherapy education. The rating a student receives is heavily dependent on the examiner the student is rated by. No significant difference in the interrater reliability between the two schools could be detected. These findings are confirmed by the two methods ICC and Krippendorff’s alpha. Besides the examiners’ assessment behaviour shows a certain in-group favouritism and central tendency bias when looking at the rating distribution. Overall, the physiotherapy students do not receive an assessment as reliable as they need to improve their skills properly. Collaborative efforts in the physiotherapy community concerning the assessment process should be made to receive more reliable measurements of physiotherapy students’ performance. Contributions and input from leading teaching institutions are necessary.

A form of standardized assessment to improve reliability, international or at least national, would be desirable. Different assessment formats as well as the use of automated, digital assessment tools could be a possibility to improve reliability in the assessment process of psychomotor skills in physiotherapy and other health care professions and should be the subject of further study.

## Supplementary Information

**Additional file 1.** ICC estimates in categories.

**Additional file 2.** Krippendorff’s alpha estimates.

**Additional file 3.** Likert plot.

**Additional file 4: Addendum A.** ICC estimates by evaluation criterion.

## Data Availability

The datasets used and/or analysed during the current study are available from the corresponding author on reasonable request.

## Abbreviations

ICC: Intraclass-correlation coefficient
mini-CEX: Mini-Clinical Evaluation Exercise
mini-PEX: Mini-Peer Evaluation Exercise
MediWeCo: Media Didactics Meets Wearable Computing
BMBF: German Federal Ministry of Education and Research
ESF: European Union Social Fund
PNF: Proprioceptive neuromuscular facilitation
AC: Schule für Physiotherapie Uniklinik RWTH Aachen
HH: Grone Berufsfachschule für Physiotherapie Hamburg
CI: 95% confidence interval
OSCE: Objective Structured Clinical Examination
APP: Assessment of Physiotherapy Practice
