# Supplementary material for: Interrater reliability in the assessment of physiotherapy students
Source: BMC Med Educ. 2022 Mar 16;22:186. doi: 10.1186/s12909-022-03231-y (PMC8928589; doi:10.1186/s12909-022-03231-y)
Supplement: Supplementary file 1 — Additional file 1. ICC estimates in categories. [file 12909_2022_3231_MOESM1_ESM.docx]

| Criteria | AC vs HH | 1,2 | 1,3 | 1,4 | 1,5 | 1,6 | 2,3 | 2,4 | 2,5 | 2,6 | 3,4 | 3,5 | 3,6 | 4,5 | 4,6 | 5,6 |
| --- | --- | --- | --- | --- | --- | --- | --- | --- | --- | --- | --- | --- | --- | --- | --- | --- |
| Bench Height | 0.627 | 0.473 | -0.155 | 0.104 | 0.357 | 0.366 | -0.359 | 0.016 | 0.423 | 0.449 | -0.069 | 0.073 | 0.259 | 0.808 | -0.132 | 0.893 |
| Treatment Area | 0.278 | -0.79 | !!! | 0 | -0.087 | !!! | -0.55 | -0.384 | -0.398 | -0.573 | 0.66 | -0.038 | -0.018 | 0.105 | -0.023 | 0.606 |
| Patient Position | 0.189 | -0.01 | -0.09 | -0.095 | 0.481 | -0.122 | -0.31 | -0.338 | 0.008 | -0.373 | 0 | 0 | -0.083 | -0.091 | 1 | -0.037 |
| Verbal Communication | 0.759 | 0.511 | 0.072 | 0.429 | 0.247 | 0.802 | 0.321 | 0.542 | 0.646 | 0.55 | 0.3 | 0.259 | 0.098 | 0.551 | 0.513 | 0.377 |
| Explanation | 0.876 | 0.876 | 0.641 | 0.729 | 0.665 | 0.722 | 0.25 | 0.465 | 0.773 | 0.776 | 0.852 | 0.162 | 0.3 | 0.577 | 0.406 | 0.83 |
| Rhythm | -0.306 | 0.137 | 0.086 | -0.259 | -0.19 | -0.005 | 0.254 | -0.231 | -0.243 | -0.279 | 0.375 | 0.08 | 0.432 | 0.222 | 0.626 | 0.661 |
| Passive | 0.169 | -0.227 | 0.025 | 0.137 | 0.324 | -0.183 | 0.296 | -0.085 | -0.173 | -0.02 | -0.03 | -0.087 | -0.431 | -0.389 | -0.135 | -0.301 |
| Active-Assistive | 0.55 | 0.651 | -0.438 | 0.554 | 0.139 | 0.673 | 0.263 | 0.69 | -0.058 | 0 | -0.212 | -0.242 | -0.254 | -0.265 | 0.663 | 0.38 |
| Resistive | 0.602 | 0.575 | -0.362 | 0.656 | -0.192 | 0.404 | 0.033 | 0.882 | 0.095 | 0.385 | -0.139 | -0.26 | -0.272 | -0.196 | 0 | -0.307 |
| Active | 0.388 | 0.293 | 0.744 | 0.879 | -0.405 | 0.632 | -0.202 | 0.48 | -0.531 | 0.165 | 0.353 | -0.562 | 0.449 | -0.194 | 0.84 | -0.634 |
| End Position | 0.226 | -0.415 | 0.733 | 0.166 | 0.072 | 0.183 | -0.148 | 0.381 | -0.335 | -0.427 | 0.257 | -0.279 | -0.007 | -0.214 | 0.094 | 0.616 |
| Diagonal | 0.182 | 0.423 | 0.555 | 0.569 | 0.202 | 0.278 | 0.376 | 0.03 | -0.243 | 0.034 | 0.265 | -0.21 | -0.194 | 0.076 | 0.631 | 0.771 |
| Movement Components | 0.234 | 0.056 | -0.11 | 0.166 | 0.411 | 0.3 | 0.07 | -0.04 | -0.201 | -0.169 | 0.209 | 0.07 | -0.065 | 0.269 | 0.07 | 0.62 |
| Timing | -1.115 | -0.067 | -0.036 | -0.036 | -0.193 | -0.126 | 0 | -0.109 | -0.383 | -0.488 | -0.058 | -0.017 | -0.074 | 0.136 | -0.257 | 0.171 |
| Body Position | 0.572 | 0.678 | 0.231 | 0.614 | 0.288 | 0.586 | 0.301 | 0.539 | -0.015 | 0.674 | 0.299 | -0.51 | -0.659 | -0.027 | 0.28 | 0.431 |
| Body Mechanics | 0.268 | 0.295 | 0.093 | -0.161 | 0.139 | 0.154 | 0.379 | 0.129 | 0.165 | 0.453 | 0.146 | 0.085 | -0.43 | 0.057 | 0.156 | 0.341 |
| Lumbrical Grip | 0.656 | 0.531 | 0.11 | 0.251 | 0.088 | 0.48 | 0.362 | 0.364 | 0.49 | 0.316 | 0.329 | 0.52 | 0.158 | 0.562 | 0.049 | 0.816 |
| Stimulus | 0.45 | 0.281 | -0.227 | -0.047 | 0.327 | 0.384 | 0.498 | -0.125 | 0.224 | -0.12 | 0.114 | 0.371 | -0.168 | 0.454 | -0.166 | 0.272 |
| Resistance | -0.238 | 0.379 | 0.061 | -0.126 | 0.006 | -0.389 | 0.201 | -0.128 | -0.161 | -0.158 | -0.254 | 0.052 | -0.247 | -0.423 | 0.124 | 0.342 |
| Overall Rating | 0.556 | 0.419 | 0.457 | 0.323 | 0.512 | 0.512 | 0.553 | 0.011 | 0.175 | 0.064 | 0.28 | 0.408 | 0.213 | 0.568 | 0.256 | 0.755 |
