# Supplementary material for: Interrater reliability in the assessment of physiotherapy students
Source: BMC Med Educ. 2022 Mar 16;22:186. doi: 10.1186/s12909-022-03231-y (PMC8928589; doi:10.1186/s12909-022-03231-y)
Supplement: Supplementary file 2 — Additional file 2. Krippendorff’s alpha estimates. [file 12909_2022_3231_MOESM2_ESM.docx]

|  |  | Krippendorff’s alpha |
| --- | --- | --- |
|  | Bench Height | 0.330 |
|  | Treatment Area | 0.009 |
|  | Patient Position | 0.06 |
|  | Verbal Communication | 0.613 |
|  | Explanation | 0.613 |
|  | Rhythm | 0.118 |
|  | Passive | -0.013 |
|  | Active Assistive | 0.204 |
|  | Resistive | 0.165 |
| All examiners | Active | 0.138 |
|  | Endposition | 0.097 |
|  | Diagonal | 0.237 |
|  | Movement Components | 0.124 |
|  | Timing | -0.079 |
|  | Bodyposition | 0.329 |
|  | Bodymechanics | 0.198 |
|  | Lumbrical Grip | 0.379 |
|  | Stimulus | 0.2 |
|  | Resistance | 0.024 |
|  | Overall Rating | 0.375 |
|  | Bench Height | 0.177 |
|  | Treatment Area | -0.344 |
|  | Patient Position | 0.014 |
|  | Verbal Communication | 0.311 |
|  | Explanation | 0.615 |
|  | Rhythm | 0.224 |
|  | Passive | -0.005 |
|  | Active Assistive | 0.081 |
|  | Resistive | 0.181 |
| AC examiners | Active | 0.397 |
|  | Endposition | 0.012 |
|  | Diagonal | 0.423 |
|  | Movement Components | -0.05 |
|  | Timing | -0.023 |
|  | Bodyposition | 0.431 |
|  | Bodymechanics | 0.283 |
|  | Lumbrical Grip | 0.35 |
|  | Stimulus | 0.269 |
|  | Resistance | 0.241 |
|  | Overall Rating | 0.48 |
|  | Bench Height | 0.713 |
|  | Treatment Area | 0.233 |
|  | Patient Position | 0.053 |
|  | Verbal Communication | 0.471 |
|  | Explanation | 0.58 |
|  | Rhythm | 0.526 |
|  | Passive | -0.271 |
|  | Active Assistive | 0.199 |
|  | Resistive | -0.091 |
| HH examiners | Active | -0.083 |
|  | Endposition | 0.197 |
|  | Diagonal | 0.563 |
|  | Movement Components | 0.296 |
|  | Timing | 0.017 |
|  | Bodyposition | 0.277 |
|  | Bodymechanics | 0.257 |
|  | Lumbrical Grip | 0.502 |
|  | Stimulus | 0.194 |
|  | Resistance | 0.093 |
|  | Overall Rating | 0.554 |
