# Supplementary material for: Interrater reliability in the assessment of physiotherapy students
Source: BMC Med Educ. 2022 Mar 16;22:186. doi: 10.1186/s12909-022-03231-y (PMC8928589; doi:10.1186/s12909-022-03231-y)
Supplement: Supplementary file 3 — Additional file 3. Likert plot. [file 12909_2022_3231_MOESM3_ESM.docx]

*Note: dark red=1 point on the rating scale red=2 points light red=3 points light blue=4 points blue=5 points dark blue=6 points*
