# Supplementary material for: Interrater reliability in the assessment of physiotherapy students
Source: BMC Med Educ. 2022 Mar 16;22:186. doi: 10.1186/s12909-022-03231-y (PMC8928589; doi:10.1186/s12909-022-03231-y)
Supplement: Supplementary file 4 — Additional file 4: Addendum A. ICC estimates by evaluation criterion. [file 12909_2022_3231_MOESM4_ESM.docx]

Addendum A

ICC estimates by evaluation criterion

| Overall Rating   \|  \| 2 \| 3 \| 4 \| 5 \| 6 \| HH \| \| --- \| --- \| --- \| --- \| --- \| --- \| --- \| \| 1 \| 0.419 \| 0.457 \| 0.323 \| 0.512 \| 0.512 \|  \| \| 2 \|  \| 0.553 \| 0.011 \| 0.175 \| 0.064 \|  \| \| 3 \|  \|  \| 0.28 \| 0.408 \| 0.213 \|  \| \| 4 \|  \|  \|  \| 0.568 \| 0.256 \|  \| \| 5 \|  \|  \|  \|  \| 0.755 \|  \| \| AC \|  \|  \|  \|  \|  \| 0.556 \| | Bench Height   \|  \| 2 \| 3 \| 4 \| 5 \| 6 \| HH \| \| --- \| --- \| --- \| --- \| --- \| --- \| --- \| \| 1 \| 0.473 \| -0.155 \| 0.104 \| 0.357 \| 0.366 \|  \| \| 2 \|  \| -0.359 \| 0.016 \| 0.423 \| 0.449 \|  \| \| 3 \|  \|  \| -0.069 \| 0.073 \| 0.259 \|  \| \| 4 \|  \|  \|  \| 0.808 \| -0.132 \|  \| \| 5 \|  \|  \|  \|  \| 0.893 \|  \| \| AC \|  \|  \|  \|  \|  \| 0.627 \| |
| --- | --- | --- | --- | --- | --- | --- | --- | --- | --- | --- | --- | --- | --- | --- | --- | --- | --- | --- | --- | --- | --- | --- | --- | --- | --- | --- | --- | --- | --- | --- | --- | --- | --- | --- | --- | --- | --- | --- | --- | --- | --- | --- | --- | --- | --- | --- | --- | --- | --- | --- | --- | --- | --- | --- | --- | --- | --- | --- | --- | --- | --- | --- | --- | --- | --- | --- | --- | --- | --- | --- | --- | --- | --- | --- | --- | --- | --- | --- | --- | --- | --- | --- | --- | --- | --- | --- | --- | --- | --- | --- | --- | --- | --- | --- | --- | --- | --- | --- | --- |
| *Treatment Area*   \|  \| 2 \| 3 \| 4 \| 5 \| 6 \| HH \| \| --- \| --- \| --- \| --- \| --- \| --- \| --- \| \| 1 \| -0.79 \| !!! \| 0 \| -0.087 \| !!! \|  \| \| 2 \|  \| -0.55 \| -0.384 \| -0.398 \| -0.573 \|  \| \| 3 \|  \|  \| 0.66 \| -0.038 \| -0.018 \|  \| \| 4 \|  \|  \|  \| 0.105 \| -0.023 \|  \| \| 5 \|  \|  \|  \|  \| 0.606 \|  \| \| AC \|  \|  \|  \|  \|  \| 0.278 \| | *Patient Position*   \|  \| 2 \| 3 \| 4 \| 5 \| 6 \| HH \| \| --- \| --- \| --- \| --- \| --- \| --- \| --- \| \| 1 \| -0.1 \| -0.09 \| -0.095 \| 0.481 \| -0.122 \|  \| \| 2 \|  \| -0.31 \| -0.338 \| 0.008 \| -0.373 \|  \| \| 3 \|  \|  \| 0 \| 0 \| -0.083 \|  \| \| 4 \|  \|  \|  \| -0.091 \| 1 \|  \| \| 5 \|  \|  \|  \|  \| -0.037 \|  \| \| AC \|  \|  \|  \|  \|  \| 0.189 \| |
| *Verbal Communication*   \|  \| 2 \| 3 \| 4 \| 5 \| 6 \| HH \| \| --- \| --- \| --- \| --- \| --- \| --- \| --- \| \| 1 \| 0.511 \| 0.072 \| 0.429 \| 0.247 \| 0.802 \|  \| \| 2 \|  \| 0.321 \| 0.542 \| 0.646 \| 0.55 \|  \| \| 3 \|  \|  \| 0.3 \| 0.259 \| 0.098 \|  \| \| 4 \|  \|  \|  \| 0.551 \| 0.513 \|  \| \| 5 \|  \|  \|  \|  \| 0.377 \|  \| \| AC \|  \|  \|  \|  \|  \| 0.759 \| | *Explanation*   \|  \| 2 \| 3 \| 4 \| 5 \| 6 \| HH \| \| --- \| --- \| --- \| --- \| --- \| --- \| --- \| \| 1 \| 0.876 \| 0.641 \| 0.729 \| 0.665 \| 0.722 \|  \| \| 2 \|  \| 0.25 \| 0.465 \| 0.773 \| 0.776 \|  \| \| 3 \|  \|  \| 0.852 \| 0.162 \| 0.3 \|  \| \| 4 \|  \|  \|  \| 0.577 \| 0.406 \|  \| \| 5 \|  \|  \|  \|  \| 0.83 \|  \| \| AC \|  \|  \|  \|  \|  \| 0.876 \| |
| *Rhythm*   \|  \| 2 \| 3 \| 4 \| 5 \| 6 \| HH \| \| --- \| --- \| --- \| --- \| --- \| --- \| --- \| \| 1 \| 0.137 \| 0.086 \| -0.259 \| -0.19 \| -0.005 \|  \| \| 2 \|  \| 0.254 \| -0.231 \| -0.243 \| -0.279 \|  \| \| 3 \|  \|  \| 0.375 \| 0.8 \| 0.432 \|  \| \| 4 \|  \|  \|  \| 0.222 \| 0.626 \|  \| \| 5 \|  \|  \|  \|  \| 0.661 \|  \| \| AC \|  \|  \|  \|  \|  \| -0.306 \| | *Passive*   \|  \| 2 \| 3 \| 4 \| 5 \| 6 \| HH \| \| --- \| --- \| --- \| --- \| --- \| --- \| --- \| \| 1 \| -0.227 \| 0.025 \| 0.137 \| 0.324 \| -0.183 \|  \| \| 2 \|  \| 0.296 \| -0.085 \| -0.173 \| -0.2 \|  \| \| 3 \|  \|  \| -0.03 \| -0.087 \| -0.431 \|  \| \| 4 \|  \|  \|  \| -0.389 \| -0.135 \|  \| \| 5 \|  \|  \|  \|  \| -0.301 \|  \| \| AC \|  \|  \|  \|  \|  \| 0.169 \| |
| *Active Assistive*   \|  \| 2 \| 3 \| 4 \| 5 \| 6 \| HH \| \| --- \| --- \| --- \| --- \| --- \| --- \| --- \| \| 1 \| 0.651 \| -0.438 \| 0.554 \| 0.139 \| 0.673 \|  \| \| 2 \|  \| 0.263 \| 0.69 \| -0.058 \| 0 \|  \| \| 3 \|  \|  \| -0.212 \| -0.242 \| -0.254 \|  \| \| 4 \|  \|  \|  \| -0.265 \| 0.663 \|  \| \| 5 \|  \|  \|  \|  \| 0.38 \|  \| \| AC \|  \|  \|  \|  \|  \| 0.55 \| | *Resistive*   \|  \| 2 \| 3 \| 4 \| 5 \| 6 \| HH \| \| --- \| --- \| --- \| --- \| --- \| --- \| --- \| \| 1 \| 0.575 \| -0.362 \| 0.656 \| -0.192 \| 0.404 \|  \| \| 2 \|  \| 0.033 \| 0.882 \| 0.095 \| 0.385 \|  \| \| 3 \|  \|  \| -0.139 \| -0.26 \| -0.272 \|  \| \| 4 \|  \|  \|  \| -0.196 \| 0 \|  \| \| 5 \|  \|  \|  \|  \| -0.307 \|  \| \| AC \|  \|  \|  \|  \|  \| 0.602 \| |

| *Active*   \|  \| 2 \| 3 \| 4 \| 5 \| 6 \| HH \| \| --- \| --- \| --- \| --- \| --- \| --- \| --- \| \| 1 \| 0.293 \| 0.744 \| 0.879 \| -0.405 \| 0.632 \|  \| \| 2 \|  \| -0.202 \| 0.48 \| -0.531 \| 0.165 \|  \| \| 3 \|  \|  \| 0.353 \| -0.562 \| 0.449 \|  \| \| 4 \|  \|  \|  \| -0.194 \| 0.84 \|  \| \| 5 \|  \|  \|  \|  \| -0.634 \|  \| \|  \|  \|  \|  \|  \|  \| 0.388 \| | *End Position*   \|  \| 2 \| 3 \| 4 \| 5 \| 6 \| HH \| \| --- \| --- \| --- \| --- \| --- \| --- \| --- \| \| 1 \| -0.415 \| 0.733 \| 0.166 \| 0.072 \| 0.183 \|  \| \| 2 \|  \| -0.148 \| 0.381 \| -0.335 \| -0.427 \|  \| \| 3 \|  \|  \| 0.257 \| -0.279 \| -0.007 \|  \| \| 4 \|  \|  \|  \| -0.214 \| 0.094 \|  \| \| 5 \|  \|  \|  \|  \| 0.616 \|  \| \| AC \|  \|  \|  \|  \|  \| 0.226 \| |
| --- | --- | --- | --- | --- | --- | --- | --- | --- | --- | --- | --- | --- | --- | --- | --- | --- | --- | --- | --- | --- | --- | --- | --- | --- | --- | --- | --- | --- | --- | --- | --- | --- | --- | --- | --- | --- | --- | --- | --- | --- | --- | --- | --- | --- | --- | --- | --- | --- | --- | --- | --- | --- | --- | --- | --- | --- | --- | --- | --- | --- | --- | --- | --- | --- | --- | --- | --- | --- | --- | --- | --- | --- | --- | --- | --- | --- | --- | --- | --- | --- | --- | --- | --- | --- | --- | --- | --- | --- | --- | --- | --- | --- | --- | --- | --- | --- | --- | --- | --- |
| *Diagonal*   \|  \| 2 \| 3 \| 4 \| 5 \| 6 \| HH \| \| --- \| --- \| --- \| --- \| --- \| --- \| --- \| \| 1 \| 0.423 \| 0.555 \| 0.569 \| 0.202 \| 0.278 \|  \| \| 2 \|  \| 0.376 \| 0.03 \| -0.243 \| 0.034 \|  \| \| 3 \|  \|  \| 0.265 \| -0.21 \| -0.194 \|  \| \| 4 \|  \|  \|  \| 0.076 \| 0.631 \|  \| \| 5 \|  \|  \|  \|  \| 0.771 \|  \| \| AC \|  \|  \|  \|  \|  \| 0.182 \| | *Movement Components*   \|  \| 2 \| 3 \| 4 \| 5 \| 6 \| HH \| \| --- \| --- \| --- \| --- \| --- \| --- \| --- \| \| 1 \| 0.056 \| -0.11 \| 0.166 \| 0.411 \| 0.3 \|  \| \| 2 \|  \| 0.07 \| -0.04 \| -0.201 \| -0.169 \|  \| \| 3 \|  \|  \| 0.209 \| 0.07 \| -0.065 \|  \| \| 4 \|  \|  \|  \| 0.269 \| 0.07 \|  \| \| 5 \|  \|  \|  \|  \| 0.62 \|  \| \| AC \|  \|  \|  \|  \|  \| 0.234 \| |
| *Timing*   \|  \| 2 \| 3 \| 4 \| 5 \| 6 \| HH \| \| --- \| --- \| --- \| --- \| --- \| --- \| --- \| \| 1 \| -0.067 \| -0.036 \| -0.036 \| -0.193 \| -0.126 \|  \| \| 2 \|  \| 0 \| -0.109 \| -0.383 \| -0.488 \|  \| \| 3 \|  \|  \| -0.058 \| -0.017 \| -0.074 \|  \| \| 4 \|  \|  \|  \| 0.136 \| -0.257 \|  \| \| 5 \|  \|  \|  \|  \| 0.171 \|  \| \| AC \|  \|  \|  \|  \|  \| -1.115 \| | *Body Position*   \|  \| 2 \| 3 \| 4 \| 5 \| 6 \| HH \| \| --- \| --- \| --- \| --- \| --- \| --- \| --- \| \| 1 \| 0.678 \| 0.231 \| 0.614 \| 0.288 \| 0.586 \|  \| \| 2 \|  \| 0.301 \| 0.539 \| -0.015 \| 0.674 \|  \| \| 3 \|  \|  \| 0.299 \| -0.51 \| -0.659 \|  \| \| 4 \|  \|  \|  \| -0.027 \| 0.28 \|  \| \| 5 \|  \|  \|  \|  \| 0.431 \|  \| \| AC \|  \|  \|  \|  \|  \| 0.572 \| |
| *Body Mechanics*   \|  \| 2 \| 3 \| 4 \| 5 \| 6 \| HH \| \| --- \| --- \| --- \| --- \| --- \| --- \| --- \| \| 1 \| 0.295 \| 0.093 \| -0.161 \| 0.139 \| 0.154 \|  \| \| 2 \|  \| 0.379 \| 0.129 \| 0.165 \| 0.453 \|  \| \| 3 \|  \|  \| 0.146 \| 0.085 \| -0.43 \|  \| \| 4 \|  \|  \|  \| 0.057 \| 0.156 \|  \| \| 5 \|  \|  \|  \|  \| 0.341 \|  \| \| AC \|  \|  \|  \|  \|  \| 0.268 \| | *Lumbrical Grip*   \|  \| 2 \| 3 \| 4 \| 5 \| 6 \| HH \| \| --- \| --- \| --- \| --- \| --- \| --- \| --- \| \| 1 \| 0.531 \| 0.11 \| 0.251 \| 0.088 \| 0.48 \|  \| \| 2 \|  \| 0.362 \| 0.364 \| 0.49 \| 0.316 \|  \| \| 3 \|  \|  \| 0.329 \| 0.52 \| 0.158 \|  \| \| 4 \|  \|  \|  \| 0.562 \| 0.049 \|  \| \| 5 \|  \|  \|  \|  \| 0.816 \|  \| \| AC \|  \|  \|  \|  \|  \| 0.656 \| |
| *Stimulus*   \|  \| 2 \| 3 \| 4 \| 5 \| 6 \| HH \| \| --- \| --- \| --- \| --- \| --- \| --- \| --- \| \| 1 \| 0.281 \| -0.227 \| -0.047 \| 0.327 \| 0.384 \|  \| \| 2 \|  \| 0.498 \| -0.125 \| 0.224 \| -0.12 \|  \| \| 3 \|  \|  \| 0.114 \| 0.371 \| -0.168 \|  \| \| 4 \|  \|  \|  \| 0.454 \| -0.166 \|  \| \| 5 \|  \|  \|  \|  \| 0.272 \|  \| \| AC \|  \|  \|  \|  \|  \| 0.45 \| | *Resistance*   \|  \| 2 \| 3 \| 4 \| 5 \| 6 \| HH \| \| --- \| --- \| --- \| --- \| --- \| --- \| --- \| \| 1 \| 0.379 \| 0.061 \| -0.126 \| 0.006 \| -0.389 \|  \| \| 2 \|  \| 0.201 \| -0.128 \| -0.161 \| -0.158 \|  \| \| 3 \|  \|  \| -0.254 \| 0.052 \| -0.247 \|  \| \| 4 \|  \|  \|  \| -0.423 \| 0.124 \|  \| \| 5 \|  \|  \|  \|  \| 0.342 \|  \| \| AC \|  \|  \|  \|  \|  \| -0.238 \| |
